# Supplementary material for: Discovery and application of insertion-deletion (INDEL) polymorphisms for QTL mapping of early life-history traits in Atlantic salmon
Source: BMC Genomics. 2010 Mar 8;11:156. doi: 10.1186/1471-2164-11-156 (PMC2838853; doi:10.1186/1471-2164-11-156)
Supplement: Additional file 2 — Information on developed 76 locus single-run INDEL panel in Atlantic salmon. Information on fluorescence labeling, primer concentrations, PCR pooling and links to alignments, INDEL motifs and GENESCAN (Burge and Karlin 1997) predictions of genes/exons are available in html format. [file 1471-2164-11-156-S2.ZIP › Additionalfile2/table11005.html]

Contig Alignment

```
Base	A B C D E F G H I J K L M N   
1	A . . . . . . . . . . . . .    
2	T . . . . . . . . . . . . .    
3	T . . . . . . . . . . . . .    
4	T . . . . . . . . . . . . .    
5	T . . . . . . . . . . . . .    
6	T . . . . . . . . . . . . .    
7	T . . . . . . . . . . . . .    
8	T . . . . . . . . . . . . .    
9	T . . . . . . . . . . . . .    
10	T . . . . . . . . . . . . .    
11	T . . . . . . . . . . . . .    
12	T . . . . . . . . . . . . .    
13	A . . . . . . . . . . . . .    
14	A . . . . . . . . . . . . .    
15	T . . . . . . . . . . . . .    
16	T . . . . . . . . . . . . .    
17	T . . . . . . . . . . . . .    
18	T . . . . . . . . . . . . .    
19	A A A A . . . A A A A . A .    
20	A A A A . . . A A A A . A .    
21	T T T T . . . T T T T . T .    
22	G G G G . . . G G G G . G .    
23	T T T T . . . T T T T . T .    
24	T T T T . . . T T T T . T .    
25	T T T T . . . T T T T . T .    
26	A A A A . . . A A A A . A .    
27	T T T T . . . T T T T . T .    
28	T T T T . . . T T T T . T .    
29	A A A A . . . A A A A . A .    
30	A A A A . . . A A A A . A .    
31	T T T T . . . T T T T . T .    
32	A A A A . . . A A A A . A .    
33	T T T T . . . T T T T . T .    
34	A A A A . . . A A A A . A .    
35	A A A A . . . A A A A . A .    
36	T T T T . . . T T T T . T .    
37	T T T T . . . T T T T . T .    
38	T T T T . . . T T T T . T .    
39	G G G G . . . G G G G . G .    
40	T T T T . . . T T T T . T .    
41	C C C C . . . C C C C . C .    
42	C C C C . . . C C C C . C .    
43	C C C C . . . C C C C . C .    
44	A A A A . . . A A A A . A .    
45	A A A A . . . A A A A . A .    
46	A A A A . . . A A A A . A .    
47	A A A A . . . A A A A . A .    
48	A A A A . . . A A A A . A .    
49	T T T T . . . T T T T . T .    
50	A A A A . . . A A A A . A .    
51	T T T T . . . T T T T . T .    
52	A A A A . . . A A A A . A .    
53	T T T T . . . T T T T . T .    
54	A A A A . . . A A A A . A .    
55	T T T T . . . T T T T . T .    
56	A A A A . . . A A A A . A .    
57	G G G G . . . G G G G . G .    
58	A A A A . . . A A A A . A .    
59	T T T T . . . T T T T . T .    
60	C C C C . . . C C C C . C .    
61	A A A A . . . A A A A . A .    
62	G G G G . . . G G G G . G .    
63	G G G G . . . G G G G . G .    
64	A A A A . . . A A A A . A .    
65	G G G G . . . G G G G . G .    
66	A A A A . . . A A A A . A .    
67	T T T T . . . T T T T . T .    
68	T T T T . . . T T T T . T .    
69	A A A A A A A A A A A A A .    
70	G G G G G G G G G G G G G .    
71	T T T T T T T T T T T T T .    
72	T T T T T T T T T T T T T .    
73	T T T T T T T T T T T T T .    
74	C C C C C C C C C C C C C .    
75	T T T T T T T T T T T T T .    
76	G G G G G G G G G G G G G .    
77	A A A A A A A A A A A A A .    
78	A A A A A A A A A A A A A .    
79	A A A A A A A A A A A A A .    
80	T T T T T T T T T T T T T .    
81	A A A A A A A A A A A A A .    
82	A A A A A A A A A A A A A .    
83	A A A A A A A A A A A A A .    
84	T T T T T T T T T T T T T .    
85	G G G G G G G G G G G G G .    
86	C C C C C C C C C C C C C .    
87	A A A A A A A A A A A A A .    
88	A A A A A A A A A A A A A .    
89	A A A A A A A A A A A A A .    
90	G G G G G G G G G G G G G .    
91	T T T T T T T T T T T T T .    
92	G G G G G G G G G G G G G .    
93	C C C C C C C C C C C C C .    
94	T T T T T T T T T T T T T .    
95	A A A A A A A A A A A A A .    
96	T T T T T T T T T T T T T .    
97	T T T T T T T T T T T T T .    
98	T T T T T T T T T T T T T .    
99	A A A A A A A A A A A A A .    
100	T T T T T T T T T T T T T .    
101	G G G G G G G G G G G G G .    
102	G G G G G G G G G G G G G .    
103	A A A A A A A A A A A A A .    
104	A A A A A A A A A A A A A .    
105	T T T T T T T T T T T T T .    
106	G G G G G G G G G G G G G .    
107	C C C C C C C C C C C C C .    
108	T T T T T T T T T T T T T .    
109	A A A A A A A A A A A A A .    
110	T T T T T T T T T T T T T .    
111	A A A A A A A A A A A A A .    
112	A A A A A A A A A A A A A .    
113	C C C C C C C C C C C C C .    
114	A A A A A A A A A A A A A .    
115	T T T T T T T T T T T T T .    
116	T T T T T T T T T T T T T .    
117	G G G G G G G G G G G G G .    
118	T T T T T T T T T T T T T .    
119	A A A A A A A A A A A A A .    
120	A A A A A A A A A A A A A .    
121	A A A A A A A A A A A A A .    
122	T T T T T T T T T T T T T .    
123	A A A A A A A A A A A A A .    
124	A A A A A A A A A A A A A .    
125	C C C C C C C C C C C C C .    
126	T T T T T T T T T T T T T .    
127	A A A A A A A A A A A A A .    
128	A A A A A A A A A A A A A .    
129	C C C C C C C C C C C C C .    
130	A A A A A A A A A A A A A .    
131	G G G A G G G G G G G G G .    
132	A A A A A A A A A A A A A .    
133	A A A A A A A A A A A A A .    
134	C C C C C C C C C C C C C .    
135	A A A A A A A A A A A A A .    
136	G G G G G G G G G G G G G .    
137	G G G G G G G G G G G G G .    
138	T T G G T T T T G T T T T .    
139	G G G G G G G G G G G G G .    
140	A A A A A A A A A A A A A .    
141	T T T T T T T T T T T T T .    
142	C C C C C C C C C C C C C .    
143	A A A A A A A A A A A A A .    
144	G G G G G G G G A G G G G .    
145	A A A A A A A A A A A A A .    
146	A A A A A A A A A A A A A .    
147	T T T T T T T T T T T T T .    
148	A A A A A A A A A A A A A .    
149	A A A A A A A A A A A A A .    
150	A A A A A A A A A A A A A .    
151	A A A A A A A A A A A A A .    
152	T T T T T T T T T T T T T .    
153	A A A A A A A A A A A A A .    
154	C C C C C C C C C C C C C .    
155	A A A A A A A A A A A A A .    
156	C C C C C C C C C C C C C .    
157	A A A A A A A A A A A A A .    
158	T T T T T T T T T T T T T .    
159	T T T T T T T T T T T T T .    
160	T T T T T T T T T T T T T .    
161	C C C C C C C C C C C C C .    
162	A A A A A A A A A A A A A .    
163	G G G G G G G G G G G G G .    
164	T T T T T T T T T T T T T .    
165	C C C C C C C C C C C C C .    
166	T T T T T T T T T T T T T .    
167	C C C C C C C C C C C C C .    
168	T T T T T T T T T T T T T .    
169	G G G G G G G G G G G G G .    
170	G G G G G G G G G G G G G .    
171	A A A A A A A A A A A A A .    
172	A A A A A A A A A A A A A .    
173	A A A A A A A A A A A A A .    
174	C C C C C C C C C C C C C .    
175	T T T T T T T T T T T T T .    
176	A A A A A A A A A A A A A .    
177	T T T T T T T T T T T T T .    
178	T T T T T T T T T T T T T .    
179	T T T T T T T T T T T T T .    
180	C C C C C C C C C C C C C .    
181	A A A A A A A A A A A A A .    
182	A A A A A A A A A A A A A A    
183	A A A A A A A A A A A A A A    
184	A A A A A A A A A A A A A A    
185	A A A A A A A A A A A A A A    
186	A A A A A A A A A A A A A A    
187	G G A A G G G G A G G G G G    
188	A A A A A A A A A A A A A A    
189	A A A A A A A A A A A A A A    
190	A A A A A A A A A A A A A A    
191	A A A A A A A A A A A A A A    
192	A A A A A A A A A A A A A A    
193	G G G G G G G G G G G G G G    
194	C C C C C C C C C C C C C C    
195	A A A A A A A A A A A A A A    
196	T T T T T T T T T T T T T T    
197	A A A A A A A A A A A A A A    
198	T T T T T T T T T T T T T T    
199	C C C C C C C C C C C C C C    
200	A A A A A A A A A A A A A A    
201	G G G G G G G G G G G G G G    
202	C C C C C C C C C C C C C C    
203	T T T T T T T T T T T T T T    
204	G G G G G G G G G G G G G G    
205	A A A A A A A A A A A A A A    
206	T T T T T T T T T T T T T T    
207	A A A A A A A A A A A A A A    
208	T T T T T T T T T T T T T T    
209	T T T T T T T T T T T T T T    
210	T T T T T T T T T T T T T T    
211	C C C C C C C C C C C C C C    
212	A A A A A A A A A A A A A A    
213	T T T T T T T T T T T T T T    
214	G G G G G G G G G G G G G G    
215	C C C C C C C C C C C C C C    
216	A A A A A A A A A A A A A A    
217	T T T T T T T T T T T T T T    
218	C C C C C C C C C C C C C C    
219	A A A A A A A A A A A A A A    
220	T T T T T T T T T T T T T T    
221	T T T T T T T T T T T T T T    
222	T T T T T T T T T T T T T T    
223	G G G G G G G G G G G G G G    
224	A A A A A A A A A A A A A A    
225	T T T T T T T T T T T T T T    
226	A A A A A A A A A A A A A A    
227	A A A A A A A A A A A A A A    
228	C C C C C C C C C C C C C C    
229	C C C C C C C C C C C C C C    
230	A A A A A A A A A A A A A A    
231	C C C C C C C C C C C C C C    
232	T T T T T T T T T T T T T T    
233	C C C C C C C C C C C C C C    
234	A A A A A A A A A A A A A A    
235	G G A G G G G G G G G G G G    
236	A A A A A A A A A A A A A A    
237	G G G G G G G G G G G G G G    
238	C C C C C C C C C C C C C C    
239	T T T T T T T T T T T T T T    
240	A A A A A A A A A A A A A A    
241	G G G G G G G G G G G G G G    
242	A A A A A A A A A A A A A A    
243	C C C C C C C C C C C C C C    
244	T T T T T T T T T T T T T T    
245	A A A A A A A A A A A A A A    
246	T T T T T T T T T T T T T T    
247	G G G G G G G G G G G G G G    
248	C C C C C C C C C C C C C C    
249	C C C C C C C C C C C C C C    
250	A A A A A A A A A A A A A A    
251	G G G G G G G G G G G G G G    
252	T T T T T T T T T T T T T T    
253	A A A A A A A A A A A A A A    
254	A A A A A A A A A A A A A A    
255	T T T T T T T T T T T T T T    
256	C C C C C C C C C C C C C C    
257	T T T T T T T T T T T T T T    
258	A A A A A A A A A A A A A A    
259	A A A A A A A A A A A A A A    
260	A A A A A A A A A A A A A A    
261	C C C C C C C C C C C C C C    
262	A A A A A A A A A A A A A A    
263	G G G G G G G G G G G G G G    
264	A A A A A A A A A A A A A A    
265	T T T A T T T T T T T T T T    
266	A A A A A A A A A A A A A A    
267	A A A A A A A A A A A A A A    
268	C C C C C C C C C C C C C C    
269	T T T T T T T T T T T T T T    
270	A A A A A A A A A A A A A A    
271	T T T T T T T T T T T T T T    
272	C C C C C C C C C C C C C C    
273	T T T T T T T T T T T T T T    
274	G G G G G G G G G G G G G G    
275	T T T T T T T T T T T T T T    
276	G G G G G G G G G G G G G G    
277	C C C C C C C C C C C T C C    
278	T T T T T T T T T T T T T T    
279	C C C C C C C C C C C C C C    
280	T T T T T T T T T T T T T T    
281	C C C C C C C C C C C C C C    
282	C C C C C C C C C C C C C C    
283	T T C T T T T T T T T T T T    
284	C C C C C C C C C C C C C C    
285	C C C C C C C C C C C C C C    
286	T T T T T T T T T T T T T T    
287	T T T T T T T T T T T T T T    
288	A A A A A A A A A A A A A A    
289	A A A A A A A A A A A A A A    
290	C C C C C C C C C C C C C C    
291	A A A A A A A A A A A A A A    
292	A A A A A A A A A A A A A A    
293	T T T T T T T T T T T T T T    
294	G G G G G G G G G G G G G G    
295	A A A A A A A A A A A A A A    
296	T T T T T T T T T T T T T T    
297	T T T T T T T T T T T T T T    
298	C C C C C C C C C C C C C C    
299	A A A A A A A A A A A A A A    
300	G G G G G G G G G G G G G G    
301	G G G G G G G G G G G G G G    
302	A A A A A A A A A A A A A A    
303	A A A A A A A A A A A A A A    
304	C C C C C C C C C C C C C C    
305	A A A A A A A A A A A A A A    
306	A A A A A A A A A A A A A A    
307	C C C C C C C C C C C C C C    
308	C C C C C C C C C C C C C C    
309	T T T T T T T T T T T T T T    
310	G G G G G G G G G G G G G G    
311	A A A A A A A A A A A A A A    
312	A A A A A A A A A A A A A A    
313	T T T T T T T T T T T T T T    
314	G G G G G G G G G G G G G G    
315	A A A A A A A A A A A A A A    
316	A A A A A A A A A A A A A A    
317	A A A A A A A A A A A A A A    
318	C C C C C C C C C C C C C C    
319	T T T T T T T T T T T T T T    
320	G G G G G G G G G G G G G G    
321	T T T T T T T T T T T T T T    
322	C C C C C C C C C C C C C C    
323	T T T T T T T T T T T T T T    
324	T T T T T T T T T T T T T T    
325	C C C C C C C C C C C C C C    
326	T T T T T T T T T T T T T T    
327	A A A A A A A A A A A A A A    
328	T T T T T T T T T T T T T T    
329	T T T T T T T T T T T T T T    
330	C C C C C C C C C C C C C C    
331	A A A A A A A A A A A A A A    
332	C C C C C C C C C C C C C C    
333	T T T T T T T T T T T T T T    
334	C C C C C C C C C C C C C C    
335	- - - - - - - T T T T - T T  6	 indel  
336	- - - - - - - T T T T - T T  6	 indel  
337	A A A A A A A A A A A A A A    
338	T T T T T T T T T T T T T T    
339	C C C C C C C C C C C C C C    
340	A A A A A A A A A A A A A A    
341	A A A A A A A A A A A A A A    
342	T T T T T T T T T T T T T T    
343	T T T T T T T T T T T T T T    
344	G G G G G G G G G G G G G G    
345	G G G G G G G G G G G G G G    
346	T T T T T T T T T T T T T T    
347	C C C C C C C C C C C C C C    
348	C C C C C C C C C C C C C C    
349	A A A A A A A A A A A A A A    
350	T T T T T T T T T T T T T T    
351	G G G G G G G G G G G G G G    
352	G G G G G G G G G G G G G G    
353	A A A A A A A A A A A A A A    
354	T T T T T T T T T T T T T T    
355	C C C C C C C C C C C C C C    
356	A A A A A A A A A A A A A A    
357	T T T T T T T T T T T T T T    
358	T T T T T T T T T T T T T T    
359	T T T T T T T T T T T T T T    
360	C C C C C C C C C C C C C C    
361	C C C C C C C C C C C C C C    
362	T T T T T T T T T T T T T T    
363	A A A A A A A A A A A A A A    
364	A A A A A A A A A A A A A A    
365	C C C C C C C C C C C C C C    
366	T T T T T T T T T T T T T T    
367	A A A A A A A A A A A A A A    
368	G G G G G G G G G G G G G G    
369	A A A A A A A A A A A A A A    
370	G G G G G G G G G G G G G G    
371	A A A A A A A A A A A A A A    
372	G G G G G G G G G G G G G G    
373	T T T T T T T T T T T T T T    
374	A A A A A A A A A A A A A A    
375	A A A A A A A A A A A A A A    
376	G G G G G G G G G G G G G G    
377	T T T T T T T T T T T T T T    
378	T T T T T T T T T T T T T T    
379	G G G G G G G G G G G G G G    
380	A A A A A A A A A A A A A A    
381	A A A A A A A A A A A A A A    
382	C C C C C C C C C C C C C C    
383	A A A A A A A A A A A A A A    
384	C C C C C C C C C C C C C C    
385	T T T T T T T T T T T T T T    
386	T T T T T T T T T T T T T T    
387	T T T T T T T T T T T T T T    
388	T T T T T T T T T T T T T T    
389	C C C C C C C C C C C C C C    
390	T T T T T T T T T T T T T T    
391	T T T T T T T T T T T T T T    
392	C C C C C C C C C C C C C C    
393	C C C C C C C C C C C C C C    
394	C C C C C C C C C C C C C C    
395	C C C C C C C C C C C C C C    
396	A A A A A A A A A A A A A A    
397	T T T T T T T T T T T T T T    
398	C C C C C C C C C C C C C C    
399	T T T T T T T T T T T T T T    
400	C C C C C C C C C C C C C C    
401	A A A A A A A A A A A A A A    
402	T T T T T T T T T T T T T T    
403	T T T T T T T T T T T T T T    
404	G G G G G G G G G G G G G G    
405	G G G G G G G G G G G G G G    
406	T T T T T T T T T T T T T T    
407	A A A A A A A A A A A A A A    
408	G G G G G G G G G G G G G G    
409	T T T T T T T T T T T T T T    
410	A A A A A A A A A A A A A A    
411	G G G G G G G G G G G G G G    
412	T T T T T T T T T T T T T T    
413	C C C C C C C C C C C C C C    
414	A A A A A A A A A A A A A A    
415	T T T T T T T T T T T T T T    
416	T T T T T T T T T T T T T T    
417	G G G G G G G G G G G G G G    
418	G G G G G G G G G G G G G G    
419	G G G G G G G G G G G G G G    
420	A A A A A A A A A A A A A A    
421	G G G G G G G G G G G G G G    
422	G G G G G G G G G G G G G G    
423	C C C G C C C C C C C C C C    
424	T T T T T T T T T T T T T T    
425	T T T T T T T T T T T T T T    
426	T T T T T T T T T T T T T T    
427	A A A A A A A A A A A A A A    
428	C C C C C C C C C C C C C C    
429	T T T T T T T T T T T T T T    
430	G G G G G G G G G G G G G G    
431	T T T T T T T T T T T T T T    
432	A A A A A A A A A A A A A A    
433	T T T T T T T T T T T T T T    
434	T T T T T T T T T T T T T T    
435	A A A A A A A A A A A A A A    
436	T T T T T T T T T T T T T T    
437	A A A A A A A A A A A A A A    
438	T T T T T T T T T T T T T T    
439	T T T T T T T T T T T T T T    
440	A A A A A A A A A A A A A A    
441	G G G G G G G G G G G G G G    
442	T T T T T T T T T T T T T T    
443	T T T T T T T T T T T T T T    
444	T T T T T T T T T T T T T T    
445	A A A A A A A A A A A A A A    
446	G G G G G G G G G G G G G G    
447	T T T T T T T T T T T T T T    
448	C C C C C C C C C C C C C C    
449	C C A A A A A A A A A A A A    
450	. . C C C C C C C C C C C C    
451	. . A A A A A A A A A A A A    
452	. . G G G G G G G G G G G G    
453	. . A A A A A A A A A A A A    
454	. . T T T T T T T T T T T T    
455	. . C C C C C C C C C C C C    
456	. . T T T T T T T T T T T T    
457	. . G G G G G G G G G G G G    
458	. . T T T T T T T T T T T T    
459	. . T T T T T T T T T T T T    
460	. . T T T T T T T T T T T T    
461	. . G G G G G G G G G G G G    
462	. . T T T T T T T T T T T T    
463	. . G G G G G G G G G G G G    
464	. . C C C C C C C C C C C C    
465	. . A A A A A A A A A A A A    
466	. . G G G G G G G G G G G G    
467	. . T T T T T T T T T T T T    
468	. . C C C C C C C C C C C C    
469	. . T T T T T T T T T T T T    
470	. . T T T T T T T T T T T T    
471	. . G G G G G G G G G G G G    
472	. . T T T T T T T T T T T T    
473	. . C C C C C C C C C C C C    
474	. . C A A A A A A A A A A A    
475	. . . C C C C C C C C C C C    
476	. . . A A A A A A A A A A A    
477	. . . T C T C T T T T T T T    
478	. . . A A A A A A A A A A A    
479	. . . T T T T T T T T T T T    
480	. . . C C C C C C C C C C C    
481	. . . A A A A A A A A A A A    
482	. . . T T T T T T T T T T T    
483	. . . T T T T T T T T T T T    
484	. . . C C C C C C C C C C C    
485	. . . C C C C C C C C C C C    
486	. . . T T T T T T T T T T T    
487	. . . C C C C C C C C C C C    
488	. . . C C C C C C C C C C C    
489	. . . A A A A A A A A A A A    
490	. . . G G G G G G G G G G G    
491	. . . G G G G G G G G G G G    
492	. . . A A A A A A A A A A A    
493	. . . T T T T T T T T T T T    
494	. . . T T T T T T T T T T T    
495	. . . T T T T T T T T T T T    
496	. . . C C C C C C C C C C C    
497	. . . A A A A A A A A A A A    
498	. . . T T T T T T T T T T T    
499	. . . G G G G G G G G G G G    
500	. . . G G G G G G G G G G G    
501	. . . G G G G G G G G G G G    
502	. . . G G G G G G G G G G G    
503	. . . A A A A A A A A A A A    
504	. . . T T T T T T T T T T T    
505	. . . T T T T T T T T T T T    
506	. . . C C C C C C C C C C C    
507	. . . T T T T T T T T T T T    
508	. . . T T T T T T T T T T T    
509	. . . T T T T T T T T T T T    
510	. . . G G G G G G G G G G G    
511	. . . T T T T T T T T T T T    
512	. . . G G G G G G G G G G G    
513	. . . A A A A A A A A A A A    
514	. . . T T T T T T T T T T T    
515	. . . A A A A A A A A A A A    
516	. . . T T T T T T T T T T T    
517	. . . T T T T T T T T T T T    
518	. . . T T T T T T T T T T T    
519	. . . G G G G G G G G G G G    
520	. . . C C C C C C C C C C C    
521	. . . C A A A C C C C A C C    
522	. . . G G G G G G G G G G G    
523	. . . G G G G G G G G G G G    
524	. . . C C C C C C C C C C C    
525	. . . A A A A A A A A A A A    
526	. . . A A A A A A A A A A A    
527	. . . A A A A A A A A A A A    
528	. . . A A A A A A A A A A A    
529	. . . A A A A A A A A A A A    
530	. . . T T T T T T T T T T T    
531	. . . G G G G G G G G G G G    
532	. . . C C C C C C C C C C C    
533	. . . T T T T T T T T T T T    
534	. . . T T T T T T T T T T T    
535	. . . G G G G G G G G G G G    
536	. . . A A A A A A A A A A A    
537	. . . T T T T T T T T T T T    
538	. . . T T T T T T T T T T T    
539	. . . T T T T T T T T T T T    
540	. . . T T T T T T T T T T T    
541	. . . G G G G G G G G G G G    
542	. . . C C C C C C C C C C C    
543	. . . A A A A A A A A A A A    
544	. . . T T T T T T T T T T T    
545	. . . G G G G G G G G G G G    
546	. . . G G G G G G G G G G G    
547	. . . C C C C C C C C C C C    
548	. . . A A A A A A A A A A A    
549	. . . A A A A A A A A A A A    
550	. . . T T T T T T T T T T T    
551	. . . A A A A A A A A A A A    
552	. . . T T T T T T T T T T T    
553	. . . G G G G G G G G G G G    
554	. . . C C C C C C C C C C C    
555	. . . A A A A A A A A A A A    
556	. . . A A A A A A A A A A A    
557	. . . T T T T T T T T T T T    
558	. . . G G G G G G G G G G G    
559	. . . A A A A A A A A A A A    
560	. . . T T T T T T T T T T T    
561	. . . T T T T T T T T T T T    
562	. . . T T T T T T T T T T T    
563	. . . T T T T T T T T T T T    
564	. . . G G G G G G G G G G G    
565	. . . T T T T T T T T T T T    
566	. . . C C C C C C C C C C C    
567	. . . C C C C C C C C C C C    
568	. . . A A A A A A A A A A A    
569	. . . A A A A C C A A A A A    
570	. . . T T T T . . T T T T T    
571	. . . T T T T . . T T T T T    
572	. . . T T T T . . T T T T T    
573	. . . G G G G . . G G G G G    
574	. . . T T T T . . T T T T T    
575	. . . C C C C . . C C C C C    
576	. . . A A A A . . A A A A A    
577	. . . C C C C . . C C C C C    
578	. . . G G G G . . G G G G G    
579	. . . G G C G . . G G G G G    
580	. . . A A . A . . A A A A A    
581	. . . A C . C . . A A A A A    
582	. . . A . . . . . A A A A A    
583	. . . T . . . . . T T T T T    
584	. . . G . . . . . G G G G G    
585	. . . C . . . . . C C C C C    
586	. . . G . . . . . G G G G G    
587	. . . C . . . . . C C C C C    
588	. . . T . . . . . T T T T T    
589	. . . G . . . . . G G G G G    
590	. . . A . . . . . A A A A A    
591	. . . C . . . . . C C C C C    
592	. . . C . . . . . C C C C C    
593	. . . A . . . . . A A A A A    
594	. . . A . . . . . A A A A A    
595	. . . G . . . . . G G G G G    
596	. . . G . . . . . G G G G G    
597	. . . G . . . . . G G G G G    
598	. . . A . . . . . A A A A A    
599	. . . A . . . . . C C A A A    
600	. . . A . . . . . . . A A A    
601	. . . . . . . . . . . A A A    
602	. . . . . . . . . . . G G G    
603	. . . . . . . . . . . T T T    
604	. . . . . . . . . . . T T T    
605	. . . . . . . . . . . T T T    
606	. . . . . . . . . . . G G G    
607	. . . . . . . . . . . T T T    
608	. . . . . . . . . . . T T T    
609	. . . . . . . . . . . G G G    
610	. . . . . . . . . . . A A A    
611	. . . . . . . . . . . C C C    
612	. . . . . . . . . . . G G G    
613	. . . . . . . . . . . T T T    
614	. . . . . . . . . . . G G G    
615	. . . . . . . . . . . T T T    
616	. . . . . . . . . . . G G G    
617	. . . . . . . . . . . G G G    
618	. . . . . . . . . . . C C C    
619	. . . . . . . . . . . T T T    
620	. . . . . . . . . . . T T T    
621	. . . . . . . . . . . G G G    
622	. . . . . . . . . . . A A A    
623	. . . . . . . . . . . T T T    
624	. . . . . . . . . . . T T T    
625	. . . . . . . . . . . G G G    
626	. . . . . . . . . . . A A A    
627	. . . . . . . . . . . A A A    
628	. . . . . . . . . . . C C C    
629	. . . . . . . . . . . C C C    
630	. . . . . . . . . . . A A A    
631	. . . . . . . . . . . T T T    
632	. . . . . . . . . . . A A A    
633	. . . . . . . . . . . A A A    
634	. . . . . . . . . . . A A A    
635	. . . . . . . . . . . A A A    
636	. . . . . . . . . . . T T T    
637	. . . . . . . . . . . G G G    
638	. . . . . . . . . . . T C C    
639	. . . . . . . . . . . . G G    
640	. . . . . . . . . . . . G G    
641	. . . . . . . . . . . . C C    
642	. . . . . . . . . . . . A A    
643	. . . . . . . . . . . . A A    
644	. . . . . . . . . . . . A A    
645	. . . . . . . . . . . . A A    
646	. . . . . . . . . . . . G G    
647	. . . . . . . . . . . . T T    
648	. . . . . . . . . . . . G G    
649	. . . . . . . . . . . . C C    
650	. . . . . . . . . . . . G G    
651	. . . . . . . . . . . . G G    
652	. . . . . . . . . . . . T T    
653	. . . . . . . . . . . . G G    
654	. . . . . . . . . . . . A A    
655	. . . . . . . . . . . . A A    
656	. . . . . . . . . . . . T T    
657	. . . . . . . . . . . . G G    
658	. . . . . . . . . . . . G G    
659	. . . . . . . . . . . . T T    
660	. . . . . . . . . . . . T T    
661	. . . . . . . . . . . . G G    
662	. . . . . . . . . . . . A A    
663	. . . . . . . . . . . . A A    
664	. . . . . . . . . . . . A A    
665	. . . . . . . . . . . . T T    
666	. . . . . . . . . . . . T T    
667	. . . . . . . . . . . . G G    
668	. . . . . . . . . . . . C C    
669	. . . . . . . . . . . . A A    
670	. . . . . . . . . . . . A A    
671	. . . . . . . . . . . . G G    
672	. . . . . . . . . . . . C C    
673	. . . . . . . . . . . . C C    
674	. . . . . . . . . . . . C C    
675	. . . . . . . . . . . . T T    
676	. . . . . . . . . . . . A A    
677	. . . . . . . . . . . . T T    
678	. . . . . . . . . . . . T T    
679	. . . . . . . . . . . . T T    
680	. . . . . . . . . . . . T T    
681	. . . . . . . . . . . . T T    
682	. . . . . . . . . . . . G G    
683	. . . . . . . . . . . . T T    
684	. . . . . . . . . . . . A A    
685	. . . . . . . . . . . . C C    
686	. . . . . . . . . . . . T T    
687	. . . . . . . . . . . . G G    
688	. . . . . . . . . . . . C C    
689	. . . . . . . . . . . . G G    
690	. . . . . . . . . . . . G G    
691	. . . . . . . . . . . . T T    
692	. . . . . . . . . . . . G G    
693	. . . . . . . . . . . . A A    
694	. . . . . . . . . . . . T T    
695	. . . . . . . . . . . . G G    
696	. . . . . . . . . . . . G G    
697	. . . . . . . . . . . . G G    
698	. . . . . . . . . . . . T T    
699	. . . . . . . . . . . . C C    
700	. . . . . . . . . . . . A A    
701	. . . . . . . . . . . . G G    
702	. . . . . . . . . . . . T T    
703	. . . . . . . . . . . . T T    
704	. . . . . . . . . . . . T T    
705	. . . . . . . . . . . . T T    
706	. . . . . . . . . . . . A A    
707	. . . . . . . . . . . . T T    
708	. . . . . . . . . . . . G G    
709	. . . . . . . . . . . . C C    
710	. . . . . . . . . . . . A A    
711	. . . . . . . . . . . . . A    
712	. . . . . . . . . . . . . T    
713	. . . . . . . . . . . . . A    
714	. . . . . . . . . . . . . A    
715	. . . . . . . . . . . . . T    
716	. . . . . . . . . . . . . A    
717	. . . . . . . . . . . . . T    
718	. . . . . . . . . . . . . T    
719	. . . . . . . . . . . . . G    
720	. . . . . . . . . . . . . C    
721	. . . . . . . . . . . . . G    
722	. . . . . . . . . . . . . A    
723	. . . . . . . . . . . . . T    
724	. . . . . . . . . . . . . G    
725	. . . . . . . . . . . . . A    
726	. . . . . . . . . . . . . T    
727	. . . . . . . . . . . . . T    
728	. . . . . . . . . . . . . T    
729	. . . . . . . . . . . . . T    
730	. . . . . . . . . . . . . A    
731	. . . . . . . . . . . . . C    
732	. . . . . . . . . . . . . T    
733	. . . . . . . . . . . . . G    
734	. . . . . . . . . . . . . T    
735	. . . . . . . . . . . . . T    
736	. . . . . . . . . . . . . T    
737	. . . . . . . . . . . . . T    
738	. . . . . . . . . . . . . A    
739	. . . . . . . . . . . . . A    
740	. . . . . . . . . . . . . G    
741	. . . . . . . . . . . . . C    
742	. . . . . . . . . . . . . A    
743	. . . . . . . . . . . . . G    
744	. . . . . . . . . . . . . A    
745	. . . . . . . . . . . . . A    
746	. . . . . . . . . . . . . A    
747	. . . . . . . . . . . . . T    
748	. . . . . . . . . . . . . A    
749	. . . . . . . . . . . . . G    
750	. . . . . . . . . . . . . T    
751	. . . . . . . . . . . . . G    
752	. . . . . . . . . . . . . C    
753	. . . . . . . . . . . . . G    
754	. . . . . . . . . . . . . G    
755	. . . . . . . . . . . . . T    
756	. . . . . . . . . . . . . G    
757	. . . . . . . . . . . . . A    
758	. . . . . . . . . . . . . T    
759	. . . . . . . . . . . . . T    
760	. . . . . . . . . . . . . G    
761	. . . . . . . . . . . . . G    
762	. . . . . . . . . . . . . T    
763	. . . . . . . . . . . . . C    
764	. . . . . . . . . . . . . A    
765	. . . . . . . . . . . . . A    
766	. . . . . . . . . . . . . A    
767	. . . . . . . . . . . . . T    
768	. . . . . . . . . . . . . T    
769	. . . . . . . . . . . . . T    
770	. . . . . . . . . . . . . G    
771	. . . . . . . . . . . . . C    
772	. . . . . . . . . . . . . A    
773	. . . . . . . . . . . . . A    
774	. . . . . . . . . . . . . G    
775	. . . . . . . . . . . . . C    
776	. . . . . . . . . . . . . C    
777	. . . . . . . . . . . . . C    
778	. . . . . . . . . . . . . T    
779	. . . . . . . . . . . . . C    
780	. . . . . . . . . . . . . G    
781	. . . . . . . . . . . . . C    
782	. . . . . . . . . . . . . A    
783	. . . . . . . . . . . . . T    
784	. . . . . . . . . . . . . A    
785	. . . . . . . . . . . . . G    
786	. . . . . . . . . . . . . T    
787	. . . . . . . . . . . . . A    
788	. . . . . . . . . . . . . T    
789	. . . . . . . . . . . . . C    
790	. . . . . . . . . . . . . G    
791	. . . . . . . . . . . . . A    
792	. . . . . . . . . . . . . G    
793	. . . . . . . . . . . . . T    
794	. . . . . . . . . . . . . T    
795	. . . . . . . . . . . . . G    
796	. . . . . . . . . . . . . C    
797	. . . . . . . . . . . . . G    
798	. . . . . . . . . . . . . A    
799	. . . . . . . . . . . . . T    
800	. . . . . . . . . . . . . C    
801	. . . . . . . . . . . . . G    
802	. . . . . . . . . . . . . C    
803	. . . . . . . . . . . . . A    
804	. . . . . . . . . . . . . G    
805	. . . . . . . . . . . . . A    
806	. . . . . . . . . . . . . A    
807	. . . . . . . . . . . . . T    
808	. . . . . . . . . . . . . C    
809	. . . . . . . . . . . . . C    
810	. . . . . . . . . . . . . T    
811	. . . . . . . . . . . . . G    
812	. . . . . . . . . . . . . G    
813	. . . . . . . . . . . . . A    
814	. . . . . . . . . . . . . G    
815	. . . . . . . . . . . . . G    
816	. . . . . . . . . . . . . G    
817	. . . . . . . . . . . . . A    
818	. . . . . . . . . . . . . C    
819	. . . . . . . . . . . . . T    
820	. . . . . . . . . . . . . G    
821	. . . . . . . . . . . . . T    
822	. . . . . . . . . . . . . C    
823	. . . . . . . . . . . . . A    
824	. . . . . . . . . . . . . T    
825	. . . . . . . . . . . . . A    
826	. . . . . . . . . . . . . T    
827	. . . . . . . . . . . . . G    
828	. . . . . . . . . . . . . G    
829	. . . . . . . . . . . . . T    
830	. . . . . . . . . . . . . C    
831	. . . . . . . . . . . . . G    
832	. . . . . . . . . . . . . T    
833	. . . . . . . . . . . . . T    
834	. . . . . . . . . . . . . G    
835	. . . . . . . . . . . . . T    
836	. . . . . . . . . . . . . C    
837	. . . . . . . . . . . . . A    
838	. . . . . . . . . . . . . T    
839	. . . . . . . . . . . . . G    
840	. . . . . . . . . . . . . C    
841	. . . . . . . . . . . . . C    
842	. . . . . . . . . . . . . A    
843	. . . . . . . . . . . . . A    
844	. . . . . . . . . . . . . A    
845	. . . . . . . . . . . . . A    
846	. . . . . . . . . . . . . C    
847	. . . . . . . . . . . . . A    
848	. . . . . . . . . . . . . G    
849	. . . . . . . . . . . . . C    
850	. . . . . . . . . . . . . A    
851	. . . . . . . . . . . . . A    
852	. . . . . . . . . . . . . A    
853	. . . . . . . . . . . . . C    
854	. . . . . . . . . . . . . A    
855	. . . . . . . . . . . . . G    
856	. . . . . . . . . . . . . A    
857	. . . . . . . . . . . . . T    
858	. . . . . . . . . . . . . C    
859	. . . . . . . . . . . . . T    
860	. . . . . . . . . . . . . G    
861	. . . . . . . . . . . . . T    
862	. . . . . . . . . . . . . G    
863	. . . . . . . . . . . . . A    
864	. . . . . . . . . . . . . C    
865	. . . . . . . . . . . . . A    
866	. . . . . . . . . . . . . T    
867	. . . . . . . . . . . . . G    
868	. . . . . . . . . . . . . G    
869	. . . . . . . . . . . . . C    
870	. . . . . . . . . . . . . T    
871	. . . . . . . . . . . . . A    
872	. . . . . . . . . . . . . G    
873	. . . . . . . . . . . . . T    
874	. . . . . . . . . . . . . A    
875	. . . . . . . . . . . . . T    
876	. . . . . . . . . . . . . T    
877	. . . . . . . . . . . . . A    
878	. . . . . . . . . . . . . T    
879	. . . . . . . . . . . . . T    
880	. . . . . . . . . . . . . T    
881	. . . . . . . . . . . . . C    
882	. . . . . . . . . . . . . C    
883	. . . . . . . . . . . . . A    
884	. . . . . . . . . . . . . G    
885	. . . . . . . . . . . . . A    
886	. . . . . . . . . . . . . T    
887	. . . . . . . . . . . . . G    
888	. . . . . . . . . . . . . T    
889	. . . . . . . . . . . . . G    
890	. . . . . . . . . . . . . T    
891	. . . . . . . . . . . . . G    
892	. . . . . . . . . . . . . T    
893	. . . . . . . . . . . . . A    
894	. . . . . . . . . . . . . A    
895	. . . . . . . . . . . . . A    
896	. . . . . . . . . . . . . A    
897	. . . . . . . . . . . . . A    
898	. . . . . . . . . . . . . G    
899	. . . . . . . . . . . . . G    
900	. . . . . . . . . . . . . C    
901	. . . . . . . . . . . . . A    
902	. . . . . . . . . . . . . G    
903	. . . . . . . . . . . . . C    
904	. . . . . . . . . . . . . T    
905	. . . . . . . . . . . . . A    
906	. . . . . . . . . . . . . T    
907	. . . . . . . . . . . . . G    
908	. . . . . . . . . . . . . G    
909	. . . . . . . . . . . . . C    
910	. . . . . . . . . . . . . A    
911	. . . . . . . . . . . . . G    
912	. . . . . . . . . . . . . A    
913	. . . . . . . . . . . . . G    
914	. . . . . . . . . . . . . G    
915	. . . . . . . . . . . . . T    
916	. . . . . . . . . . . . . T    
917	. . . . . . . . . . . . . T    
918	. . . . . . . . . . . . . C    
919	. . . . . . . . . . . . . T    
920	. . . . . . . . . . . . . C    
921	. . . . . . . . . . . . . C    
922	. . . . . . . . . . . . . C    
923	. . . . . . . . . . . . . T    
924	. . . . . . . . . . . . . C    
925	. . . . . . . . . . . . . T    
926	. . . . . . . . . . . . . A    
927	. . . . . . . . . . . . . T    
928	. . . . . . . . . . . . . G    
929	. . . . . . . . . . . . . G    
930	. . . . . . . . . . . . . G    
931	. . . . . . . . . . . . . C    
932	. . . . . . . . . . . . . T    
933	. . . . . . . . . . . . . T    
934	. . . . . . . . . . . . . A    
935	. . . . . . . . . . . . . T    
936	. . . . . . . . . . . . . C    
937	. . . . . . . . . . . . . A    
938	. . . . . . . . . . . . . G    
939	. . . . . . . . . . . . . G    
940	. . . . . . . . . . . . . A    
941	. . . . . . . . . . . . . A    
942	. . . . . . . . . . . . . G    
943	. . . . . . . . . . . . . T    
944	. . . . . . . . . . . . . A    
945	. . . . . . . . . . . . . A    
946	. . . . . . . . . . . . . C    
947	. . . . . . . . . . . . . T    
948	. . . . . . . . . . . . . T    
949	. . . . . . . . . . . . . C    
950	. . . . . . . . . . . . . A    
951	. . . . . . . . . . . . . C    
952	. . . . . . . . . . . . . A    
953	. . . . . . . . . . . . . T    
954	. . . . . . . . . . . . . G    
955	. . . . . . . . . . . . . T    
956	. . . . . . . . . . . . . C    
957	. . . . . . . . . . . . . A    
958	. . . . . . . . . . . . . G    
959	. . . . . . . . . . . . . T    
960	. . . . . . . . . . . . . G    
961	. . . . . . . . . . . . . C    
962	. . . . . . . . . . . . . T    
963	. . . . . . . . . . . . . A    
964	. . . . . . . . . . . . . T    
965	. . . . . . . . . . . . . C    
966	. . . . . . . . . . . . . T    
967	. . . . . . . . . . . . . G    
968	. . . . . . . . . . . . . A    
969	. . . . . . . . . . . . . A    
970	. . . . . . . . . . . . . T    
971	. . . . . . . . . . . . . A    
972	. . . . . . . . . . . . . C    
973	. . . . . . . . . . . . . C    
974	. . . . . . . . . . . . . A    
975	. . . . . . . . . . . . . G    
976	. . . . . . . . . . . . . T    
977	. . . . . . . . . . . . . G    
978	. . . . . . . . . . . . . C    
979	. . . . . . . . . . . . . T    
980	. . . . . . . . . . . . . C    
981	. . . . . . . . . . . . . G    
982	. . . . . . . . . . . . . C    
983	. . . . . . . . . . . . . T    
984	. . . . . . . . . . . . . G    
985	. . . . . . . . . . . . . A    
986	. . . . . . . . . . . . . C    
987	. . . . . . . . . . . . . C    
988	. . . . . . . . . . . . . T    
989	. . . . . . . . . . . . . A    
990	. . . . . . . . . . . . . G    
991	. . . . . . . . . . . . . C    
992	. . . . . . . . . . . . . A    
993	. . . . . . . . . . . . . T    
994	. . . . . . . . . . . . . C    
995	. . . . . . . . . . . . . T    
996	. . . . . . . . . . . . . G    
997	. . . . . . . . . . . . . G    
998	. . . . . . . . . . . . . T    
999	. . . . . . . . . . . . . G    
1000	. . . . . . . . . . . . . C    
1001	. . . . . . . . . . . . . C    
1002	. . . . . . . . . . . . . G    
1003	. . . . . . . . . . . . . T    
1004	. . . . . . . . . . . . . T    
1005	. . . . . . . . . . . . . T    
1006	. . . . . . . . . . . . . G    
1007	. . . . . . . . . . . . . G    
1008	. . . . . . . . . . . . . T    
1009	. . . . . . . . . . . . . A    
1010	. . . . . . . . . . . . . A    
1011	. . . . . . . . . . . . . C    
1012	. . . . . . . . . . . . . C    
1013	. . . . . . . . . . . . . C    
1014	. . . . . . . . . . . . . C
```
